# Low temperature increases adenovirus replication via intracellular alkalization

**DOI:** 10.3389/fcimb.2025.1648576

**Published:** 2025-10-08

**Authors:** Wenwu Sun, Zhuang Ma, Jianping Cao, Junli Zhang

**Affiliations:** Department of Respiratory Medicine, General Hospital of Northern Theatre Command, Shenyang, China

**Keywords:** low temperature, adenovirus replication, intracellular alkalization, glycolysis, the common cold

## Abstract

Changes in environmental temperature contribute to a higher incidence of respiratory tract viral infections during the colder months of the year. However, the effect of low temperature on the replication of viruses in pulmonary epithelial cells is still elusive. In this work, we measured the change of intracellular pH (pH_i_) and the replication of adenovirus in A549 cells. We observed that exposure of cells to a cooler temperature (33°C) resulted in increases in both intracellular pH and adenovirus replication. In addition, the enhanced replication of adenovirus induced by 33°C was attenuated by inhibition of glycolysis with either 2-deoxy-D-glucose (2-DG) or PFK158. Moreover, oligomycin, which stimulates the glycolytic flux, led to a significant increase in viral replication at 37°C. Further experiments showed that low-temperature-promoted virus replication and intracellular alkalization were efficiently inhibited by the acidification of the extracellular medium. Taken together, these data suggest that intracellular alkalization and glycolysis caused by low temperature enhance adenovirus replication in host cells.

## Introduction

Acute exposure to cold temperatures contributes to the onset of the common cold (Eccles, 2020). Low temperatures also worsen cold symptoms and even lead to respiratory morbidity and mortality, with increased burden on society in terms of health services and hospital admissions during the winter season. The common cold is primarily caused by respiratory tract infections from a broad variety of respiratory viruses, such as adenovirus, parainfluenza virus, rhinovirus, respiratory syncytial virus, enterovirus, coronavirus, and influenza virus (Eccles, 2020; Heikkinen and Jävinen, 2003).

Human adenovirus (HAdV) is one of the highly contagious respiratory viruses that can result in epidemics of seasonal infections of the upper and lower respiratory tract. Multiple HAdV species, including species B, C, and E, account for 5.8%–13% of patients with acute respiratory infections. Human adenovirus type 55 (HAdV-55) belongs to species B, which was first isolated from Shanxi Province in 2006. HAdV-55 has spread widely in China during the period between 2006 and 2016 (Mao et al., 2022). The seasonal frequency of HAdV infection varies throughout the year. As the temperature rises, the number of HAdV-related pneumonia cases tends to decrease. In contrast, a higher frequency of HAdV infections happens during winter and early spring (Pscheidt et al., 2021).

Many respiratory viruses, including human adenovirus, cytomegalovirus, SARS-CoV-2, rhinovirus, and Epstein–Barr virus, reprogram host cell metabolism to promote glycolysis for their replicative advantage. The metabolic alterations produce the main energy and a carbon source for the synthesis of nucleotides, amino acids, and lipids to meet the needs of the virus for survival and reproduction (Awad et al., 2022; Allen et al., 2022). Further research studies revealed that the varying productivities of viruses are associated with virus-type specificity and species- and time-related metabolic patterns of infected host cells under different conditions (Allen et al., 2022; Awad et al., 2025). For instance, high glucose alters the glycolytic pattern, which increases SARS-CoV-2 replication in monocytes and decreases influenza and parainfluenza productivity in A549 cells, respectively (Codo et al., 2020; Awad et al., 2025). It has been reported that low temperature leads to intracellular pH elevation and subsequently enhances glycolysis (Fang et al., 2017). However, the effect of cold-induced intracellular alkalization and glycolysis on adenovirus replication in the host cell is still unclear. In the present study, we investigated the role of the change of pH_i_ induced by low temperature in the replication of HAdV-55.

## Materials and methods

### Reagents

The pH-sensitive fluorescent probe BCECF-AM (2′,7′-bis-(2-carboxyethyl)-5-carboxyfluorescein-acetoxymethyl ester) (Cat#51012) was purchased from Biotium Inc. (Hayward, CA, USA). Mouse monoclonal adenovirus hexon protein antibody (sc-80671) and FITC-conjugated m-lgG_k_ BP (sc-516140) were purchased from Santa Cruz Biotechnology (Shanghai) Co., Ltd. 2-Deoxy-D-glucose (2-DG, CAS 154-17-6) and oligomycin (CAS 1404-19-9-17-6) were purchased from MedChemExpress LLC (Shanghai), and PFK158 (Cat#S8807) was purchased from Selleckchem (Shanghai, China). The other agents were of analytical grade.

### Cell line and virus

The A549 cell, a human alveolar epithelial cell line, was purchased from the National Collection of Authenticated Cell Culture (Shanghai, China). The cell was grown in Dulbecco’s modified Eagle’s medium supplemented with 10% fetal bovine serum (pH 7.2). Human adenovirus-55 was kindly provided by Professor Hao Ren from the Naval Medical University (Shanghai, China). The virus was propagated and stored according to previously described methods (Hashimoto et al., 1991). The infectivity titer of the stock adenovirus was 2 × 10^7^ PFU/mL, and the adenovirus was stored at −80°C.

### Measurement of ECAR and pH_i_ by BCECF fluorescence

To examine the extracellular acidification rate (ECAR), A549 cells were seeded on 6-cm cell culture dishes at a density of 2.5 × 10^6^/dish for 24 h. Then, cells were incubated in 10 mL of DMEM with 10% fetal bovine serum at 37°C and 33°C for 2 h, respectively. The pH of the culture medium was measured using a pH meter. The ECAR was calculated with the change of media pH.

The pH_i_ was measured using the BCECF-AM. A549 cells were seeded in six-well dishes at a density of 2.5 × 10^5^ cells per well and were cultured in DMEM with 10% fetal bovine serum at 37°C for 1 day. The cells were incubated at different temperatures (37°C, 33°C) and extracellular pH values (pH 6.4, pH 7.2), respectively. The medium pH was adjusted with HCl. After cells were treated for 20 min with 5 µM of BCECF-AM, the cells were then rinsed to fully remove the dye. Fluorescence (excitation wavelength, 488 nm; emission wavelength, 510 nm) was measured using a fluorescence microscope (Olympus IX53, Tokyo, Japan). The cell images were recorded by a cooled CCD. Fluorescence images were repeated at least three times. For quantization, the area of the cell was selected, and the mean fluorescence intensity of BCECF probe images was determined. A calibration was performed using nigericin (10 µg/mL) in a buffer solution (140 mM of KCl, 1 mM of MgCl_2_, 1 mM of CaCl_2_, 5 mM of glucose, and 15 mM of HEPES) at fixed pH values of 7.2 and 7.6, respectively.

### Immunohistochemistry

Six coverslips (22 mm × 22 mm) were plated in 10-cm cell culture dishes. After the cells were cultured in DMEM with 10% fetal bovine serum at 37°C and 5% CO_2_, the adhered cells were incubated with adenovirus at a multiplicity of infection (MOI) of 5 for 1 h. The cells were then washed three times with phosphate-buffered saline (PBS). This operation ensures the same number of virions in each cell as much as possible. Each cover glass with cells was allowed to culture for 30 h under different conditions. The cells were fixed with 3.7% PFA for 30 min. After three washes with PBS, the cells were subjected to 0.1% Triton X-100 for cell membrane perforation. After treatment with protein block for 30 min, the cells were stained with a mouse monoclonal antibody against the adenovirus hexon protein overnight at 4°C. After three washes with TBS, FITC-conjugated m-lgG_k_ BP was added and incubated for 90 min at room temperature. After removing the excess of fluorescence-conjugated protein, the cell nuclei were stained with Hoechst 33342. The fluorescence intensity of the adenovirus hexon protein was analyzed with ImageJ software.

### Quantitative RT-PCR

Total RNA was extracted using TRIzol reagent (TaKaRa, Dalian, China) as the lysis buffer. Complementary DNA (cDNA) was then synthesized using PrimeScript RT reagent Kit (TaKaRa, Dalian, China), and subsequently, quantitative RT-PCR was performed. All reaction components were obtained from the same source (TaKaRa biotechnology, Dalian, China). The mRNA expression levels were normalized to the housekeeping gene GAPDH. The primers used were as follows: PFKFB3 F: 5′-CTGCAGAGGAGATGCCCTAC-3′, R: 5′-AGGTCCCTTCTTTGCATCCT-3′CATCCT-3′; and GAPDH F: 5′-CCACCCATGGCAAATTCCATGGCA-3′, R: 5′-TCTAGACGGCAGGTCAGGTCCACC-3′.

### Statistical analysis

All data were presented as the means ± standard error of the mean (SEM). Student’s *t*-test was used for comparison between the two groups. A *P*-value <0.05 was considered statistically significant.

## Results

### Low temperature enhances the HAdV replication

**Figure 1:**
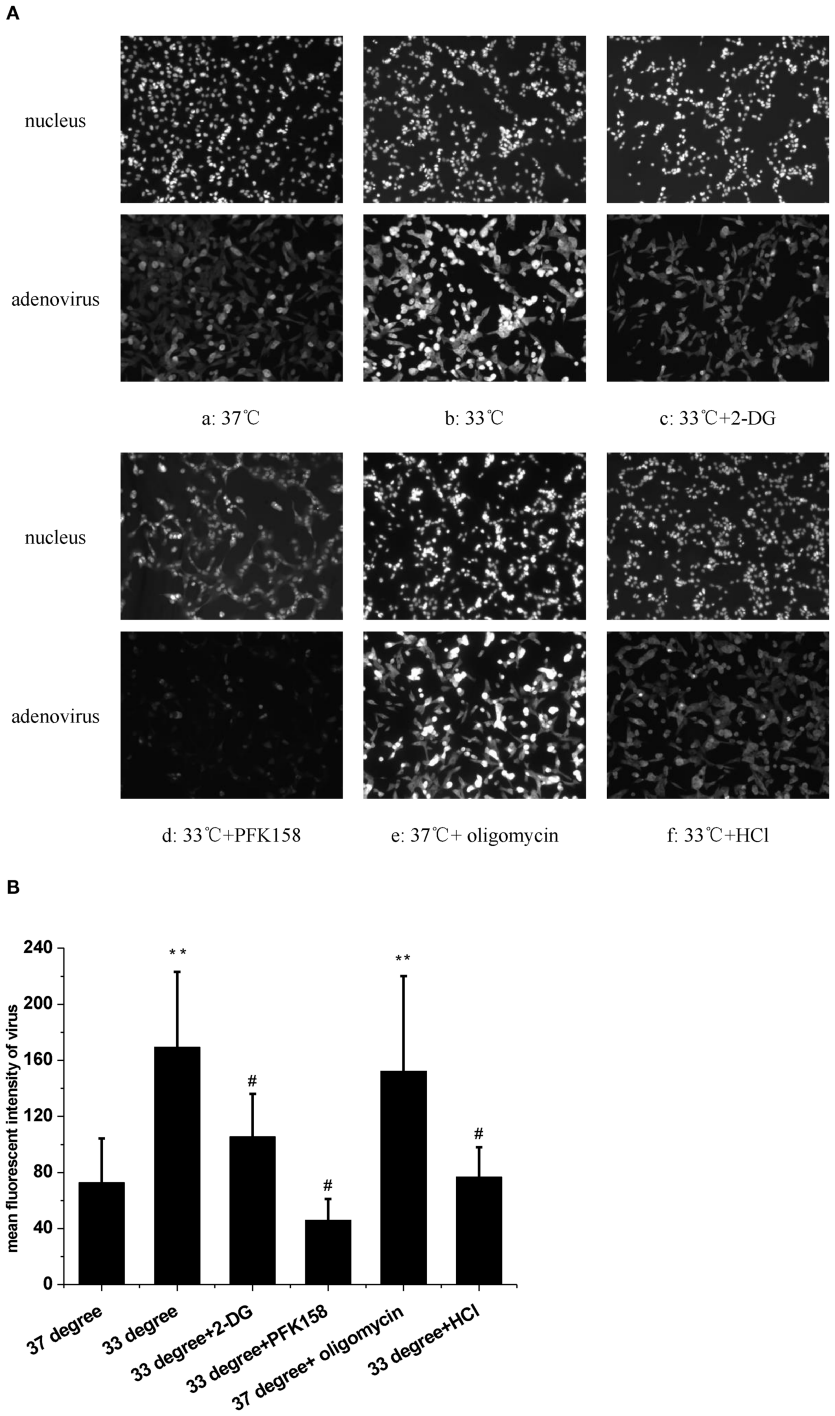
The effect of temperature and extracellular pH on adenovirus replication. **(A)** The fluorescence images of adenovius in A549 cells. (a) 37°C. (b) 33°C. (c) 33°C and 2-DG. (d) 33°C and PFK158. (e) 37°C and oligomycin. (f) 33°C and acide medium. **(B)** Statistical analysis of the replication of adenovirus in multiple experiments. **P < 0.01, cold and oligomycin increased the adenovirus replication. #*P* < 0.01, Acide medium, 2-DG and PFK158 reduced the replication of adenovirus in A549 cells at 33°C.

We first examined the effect of low temperature on HAdV replication in A549 cells. The fluorescence intensity of HAdV in a single cell was detected using immunohistochemistry. The fluorescence images showed that low temperature (33°C) significantly increased HAdV replication in cells (**Figure 1A**a,b). Statistical analysis of multicellular data indicated that HAdV replication was significantly higher in the host cells incubated at 33°C than in the host cells incubated at 37°C (**Figure 1B**, n = 30, *P* < 0.01). These observations confirmed that low temperature increases the HAdV replication in A549 cells.

### The low temperature-induced HAdV replication via glycolysis

**Figure 2:**
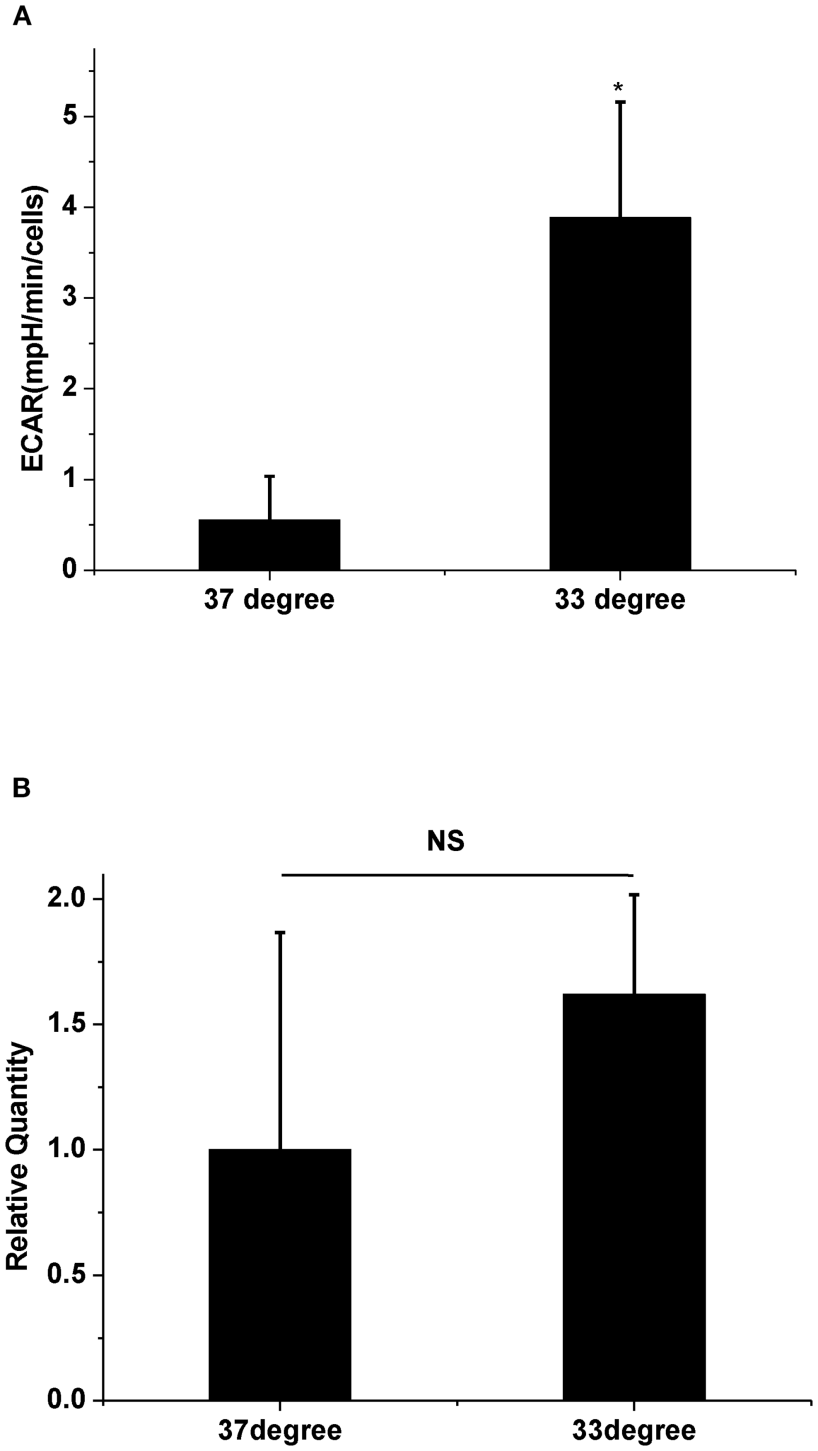
The effect of temperature on ECAR and PFKFB3 mRNA expression. **(A)** Statistical analysis of ECAR at 37°C and 33°C, respectively. * *P*<0.05 (n=3), 33°C increased ECAR compared with 37°C. **(B)** Statistical analysis of PFKFB3 mRNA expression at 37°C and 33°C, respectively. P>0.05 (n=3), There are no difference in mRNA expression of PFKFB3 between 37°C and 33°C.

We next assessed glycolytic function by measuring ECAR at 33°C and 37°C. Results indicated that 33°C significantly promoted ECAR of A549 compared to 37°C (**Figure 2A**, n = 3, *P* < 0.05), which confirmed that low temperature enhanced glycolysis. To further determine if the glycolysis pathway could affect HAdV replication at low temperature, two inhibitors of glycolysis were used. Results showed that both 5 mM of 2-DG and 2.5 μM of PFK158 obviously prevented the low temperature-induced enhancement of viral replication, respectively (**Figures 1A**c, d, **B**, n = 30, *P* < 0.01). In order to further confirm the importance of glycolysis in HAdV replication, we used oligomycin to inhibit ATP synthesis and to promote glycolysis at core body temperature (37°C). The results showed that 1 µM of oligomycin remarkably enhanced the HAdV replication at 37°C (**Figures 1A**e, **B**, n = 30, *P* < 0.01). The above results indicate that glycolytic activation is responsible for the enhanced replication of HAdV at low temperature.

### HAdV replication is dependent on intracellular alkalization

**Figure 3:**
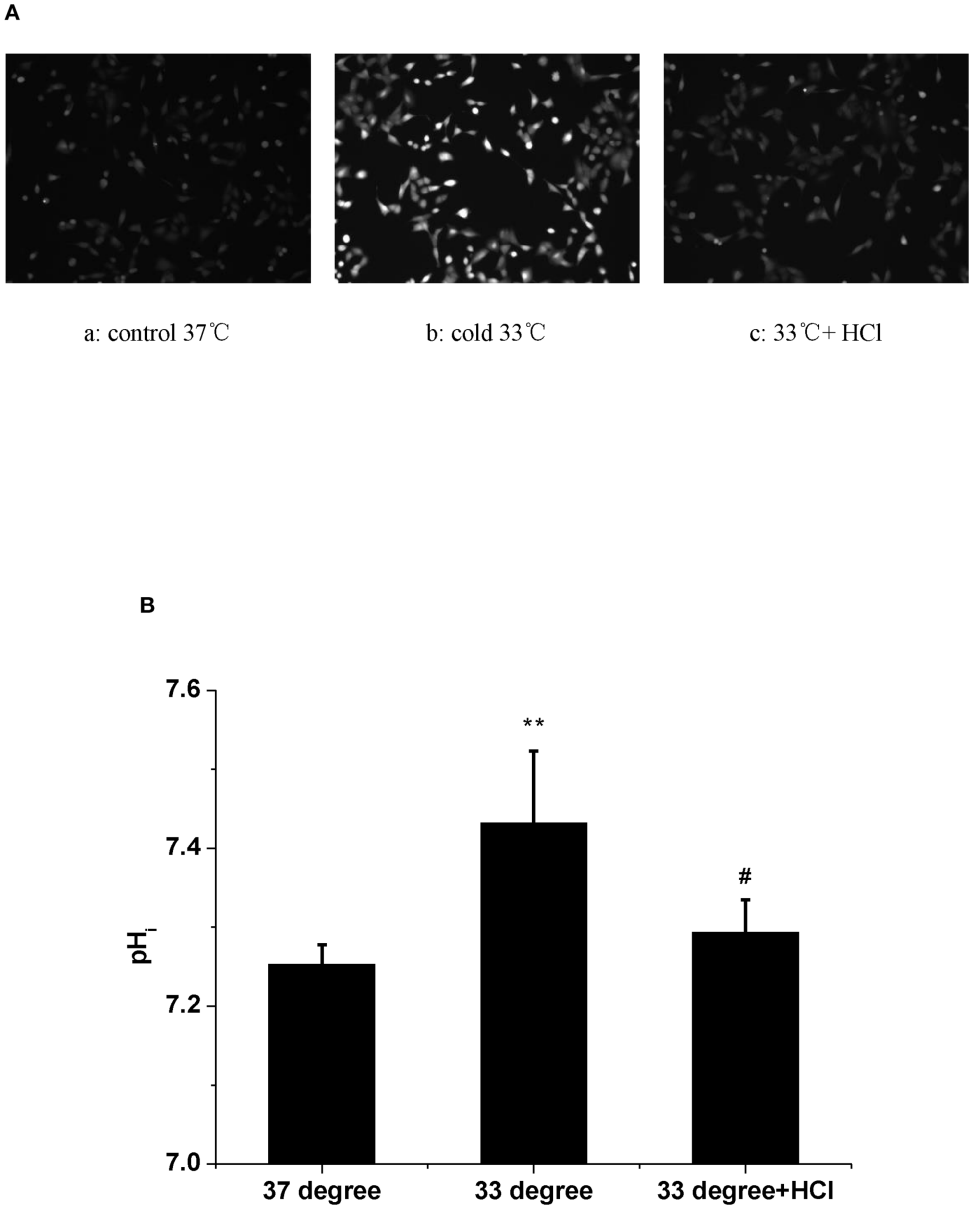
The effect of temperature and extracellular pH on intracellular pH in A549 cells. **(A)** The fluorescence images of pH_i_ in A549 cells. Fluorescence images are shown with the fluorescence intensity representing pHi. (a) 37°C. (b) 33°C. (c) 37°C and acide medium. **(B)** Statistical analysis of the values of pHi in A549 cells. **P < 0.01, Cold increased pHi compared with control. # *P* < 0.01, Acid medium decreased the elevated pHi in A549 cells, respectively.

At last, the change of pH_i_ was examined at a low-temperature condition. The results showed that exposure to a 33°C medium for 1 h induced a significant increase in the fluorescence of pH_i_ (**Fgures 3A**a,b, **B**, *n* = 30, *P* < 0.01). In view of the evidence that low temperature increases both pH_i_ and HAdV replication, the extracellular pH (pH_o_) was shifted from 7.2 to 6.4 in the next experiment. Results showed that an acidic medium largely blocked the enhanced pH_i_ caused by 33°C in A549 cells (**Figures 3A**c, **B**, *n* = 30, *P* < 0.01). At the same time, the acidic medium almost completely attenuated the cold-induced elevation of HAdV replication (**Figures 1Af, B**, n = 30, *P* < 0.01). Statistical results in **Figures 1B** and **3B** confirmed the suppressive effect of the acidic medium on the increases in both pH_i_ and the replication of HAdV at 33°C. The above results suggested that intracellular alkalization induced by low temperature is necessary for HAdV replication in A549 cells.

## Discussion

Temperature sensitivity is an important influencing factor in acute viral respiratory infections. The present explanation is that cold exposure causes vasoconstriction, which impairs extracellular vesicle swarm-mediated nasal antiviral immunity (Eccles, 2020; Huang et al., 2023). However, the mechanism underlying the association between a cooler temperature and higher virus replication still remains obscure. The possible reasons behind this phenomenon are multifaceted.

It has been reported that the recombinant adenovirus yield is higher at 32°C–35°C than at 37°C in human embryonic kidney cells (Jardon and Garnier, 2003). Airway epithelial cells are not only central to the defense against respiratory viruses but also the main hosts for respiratory viruses (Vareille et al., 2011). Here, our results confirm that low temperature promotes HAdV replication in A549 cells. Therefore, the inhalation of cold air possibly creates a cooler temperature advantage in respiratory epithelial cells for virus replication.

Metabolic reprogramming of host cells is critical in viral infections. Virus reprograms the host cell metabolism to preferentially use glycolysis as a rapid energy source and the synthesis of amino acids, lipids, and nucleotides, which contributes to virus multiplication (Thai et al., 2014; Allen et al., 2022). In this study, after the enhancement of glycolysis in A549 cells at 33°C has been confirmed, we evaluated the impact of glycolysis on virus replication under low-temperature conditions. 2-DG is an inhibitor of glycolysis. Previous studies have demonstrated that 2-DG inhibits SARS-CoV-2 replication in host cells (Bojkova et al., 2020; Codo et al., 2020). We observed the effect of 2-DG on HAdV replication at 33°C. Additionally, the 6-phosphofructo-2-kinase/fructose-2,6-bisphosphatase 3 (PFKFB3) is a critical enzyme that controls the glycolytic flux. PFK158, a specific inhibitor of PFKFB3 (Sarkar Bhattacharya et al., 2022), has been used at 33°C to further clarify the involvement of glycolysis. On the other hand, oligomycin, a glycolysis stimulator (Mirveis et al., 2024), specifically blocks proton conductance through the mitochondrial inner membrane, which contributes to mitochondrial dysfunction and glycolysis (Pagliarani et al., 2013). Oligomycin can increase SARS-CoV-2 replication in host cells through the glycolysis pathway (Codo et al., 2020). Here, oligomycin was used as a positive control at 37°C. Both results of the inhibitory effect of the two glycolysis inhibitors at 33°C and the promotion effect of the glycolysis stimulator on virus replication at 37°C would suggest that metabolic reprogramming of the host cell contributes to adenovirus replication at low-temperature conditions. Thus, low temperature may induce a metabolic advantage for airway viral infections, which could explain the higher frequency of virus infection in cold environments.

Some regulatory enzymes are involved in the non-oxidative branch of the pentose phosphate pathway (PPP) after viral infections. For example, in the non-oxidative PPP, the transketolase enzyme (TKT) converts ribose-5-phosphate and xylulose-5-phosphate to sedoheptulose 7-phosphate and glyceraldehyde 3-phosphate. Glyceraldehyde 3-phosphate is an essential intermediate in the glycolytic pathway (Guo et al., 2024). Another key glycolysis-related enzyme is transaldolase 1 (TALDO1), which produces erythrose 4-phosphate and fructose 6-phosphate with sedoheptulose 7-phosphate and glyceraldehyde 3-phosphate (Lou et al., 2024). Both regulatory enzymes are increased in the SARS-CoV-2-infected host cells (Bojkova et al., 2021). The human adenovirus protein early region 1A (E1A) also promotes the expression of several glycolytic genes, including 6-phosphogluconolactonase (6PGL), which converts 6-phosphoglucono-δ-lactone into 6-phosphogluconate (Prusinkiewicz et al., 2020). Because the cold-induced enhancement of virus replication was markedly attenuated by the PFKFB3 inhibitor (**Figure 1A**d), we next assessed whether PFKFB3 expression was upregulated at 33°C. However, the results in **Figure 2B** showed that the expression of the PFKFB3 mRNA level did not increase under cold conditions. These results suggest that the cold-induced increase in viral replication depends on glycolytic activation mediated through pathways other than upregulation of the rate-limiting enzyme PFKFB3. It is possible that alternative mechanisms enhance glycolytic flux under cold stress. Previous studies have demonstrated that low temperature increases pH_i_ and subsequently enhances glycolysis (Fang et al., 2017; Do et al., 1996). Intracellular alkalization not only directly stimulates the activity of several glycolytic enzymes, including TKT, TALDO1, and 6PGL (Alfarouk et al., 2020; Russell et al., 2022), but also rapidly induces Smad5 protein nucleocytoplasmic shuttling leading to glycolysis (Fang et al., 2017). Based on the above viewpoints, it is reasonable to speculate that cold-induced high pH_i_ could enhance HAdV replication in host cells. To investigate the effect of the change of pH_i_ on the enhanced HAdV replication at 33°C, the pH_i_ was assessed. The results showed that low temperature rapid increases pH_i_ at 1 h, which can be decreased with an acidic medium. In fact, similar changes of pH_i_ have also been observed even after 30 h (data not shown). The decreasing pH_i_ also obviously inhibits low-temperature-promoted virus replication. These results suggest that intracellular alkalization is a critical factor that enhances HAdV replication in host cells under a cold environment.

**Figure 4:**
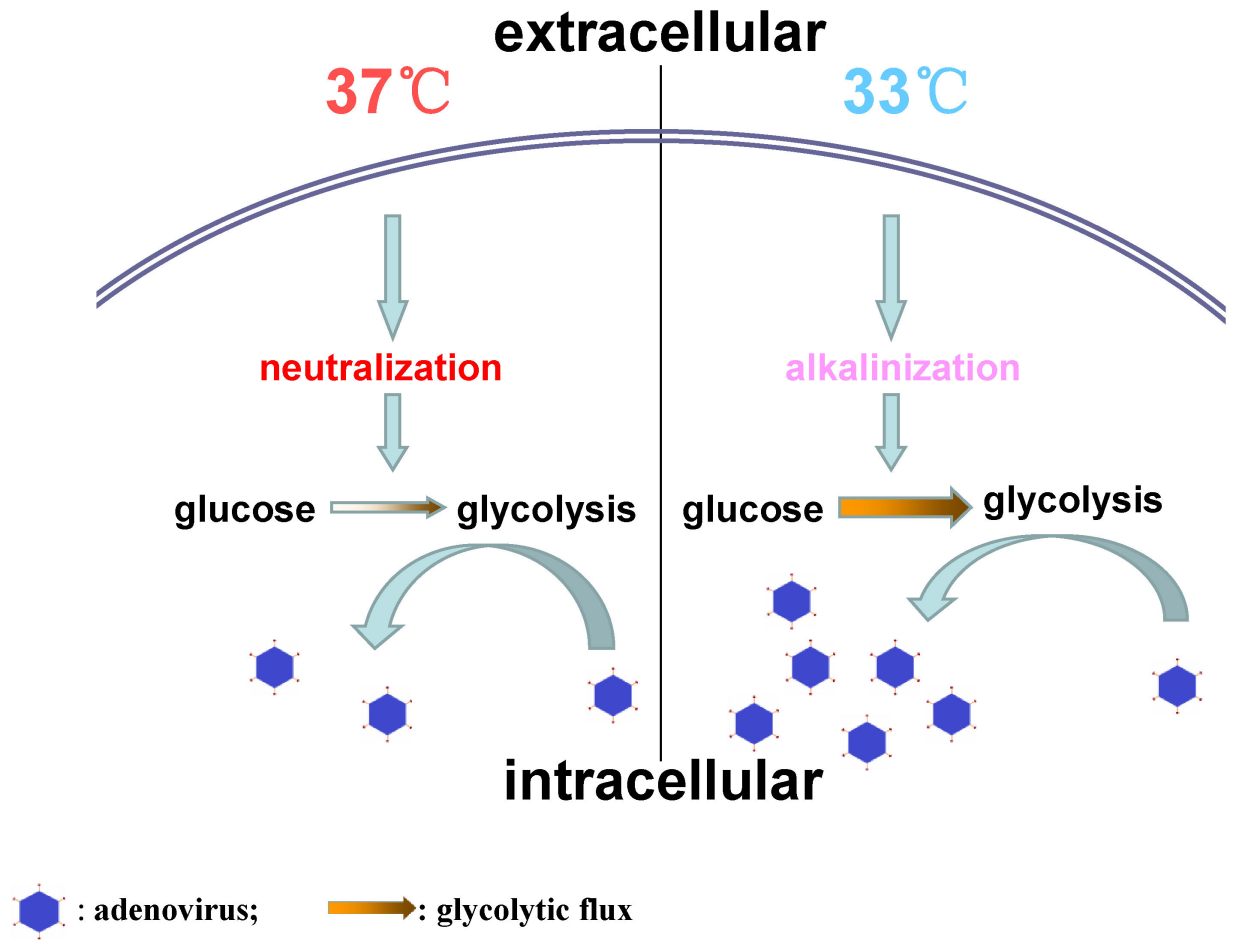
The difference of adenovirus replication between 37°C and 33°C.

In conclusion, this study demonstrates that low temperature facilitates HAdV replication in host cells through promoting intracellular alkalization and glycolysis (**Figure 4**). These findings may provide a potential explanation for the clinical phenomena that cold temperature increases respiratory virus infection prevalence, morbidity, and mortality during the winter period. Perhaps, this work might be helpful for a better understanding of the pathogenic process of “catching a cold.”

## Data Availability

The original contributions presented in the study are included in the article/supplementary material. Further inquiries can be directed to the corresponding authors.
